# Insulin-like Growth Factor 1 Impact on Alzheimer’s Disease: Role in Inflammation, Stress, and Cognition

**DOI:** 10.3390/cimb47040233

**Published:** 2025-03-27

**Authors:** Jonathan Zegarra-Valdivia, Harold Arana-Nombera, Leandro Perez-Fernandez, Milagros del Rocío Casimiro, Viviana Gallegos-Manayay, María del Rosario Oliva-Piscoya, Reyna Alamo-Medina, Eduardo Abanto-Saldaña, María Celinda Cruz-Ordinola, Carmen Paredes-Manrique, Brenda Chino-Vilca

**Affiliations:** 1 Facultad de Ciencias de la Salud, Universidad Señor de Sipán, Chiclayo 14000, Peru; anomberaharolda@uss.edu.pe (H.A.-N.); pfernandezleand@uss.edu.pe (L.P.-F.); caranamilagroro@uss.edu.pe (M.d.R.C.); gmanayayviviana@uss.edu.pe (V.G.-M.); opiscoyamariadr@uss.edu.pe (M.d.R.O.-P.); amedinareynaeli@uss.edu.pe (R.A.-M.); asaldanaeduardo@uss.edu.pe (E.A.-S.); mccruzo@uss.edu.pe (M.C.C.-O.); 2 Facultad de Humanidades, Universidad Tecnológica del Perú, Lima 15046, Peru; c27709@utp.edu.pe; 3 Achucarro Basque Center for Neuroscience, 48940 Leioa, Spain; brenda.chino@achucarro.org

**Keywords:** Alzheimer’s disease, insulin-like growth factor 1, stress, cognitive functions, neuroinflammation

## Abstract

Alzheimer’s disease (AD) is a leading cause of dementia, characterized by multifactorial interactions involving genetic, inflammatory, and metabolic dysregulation. Insulin-like growth factor 1 (IGF-I) plays a critical role in maintaining brain homeostasis through neurogenesis, synaptogenesis, and neuroprotection. However, disruptions in IGF-I signaling have been implicated in hallmark AD processes such as beta-amyloid accumulation, glucose metabolism disturbances, oxidative stress, chronic inflammation, and neuronal death. This review aims to comprehensively analyze the mechanisms by which IGF-I influences AD pathology, emphasizing its potential as both an early detection biomarker and a therapeutic target. By synthesizing clinical and preclinical study findings, we explore how chronic stress, systemic inflammation, and lifestyle factors disrupt IGF-I pathways, accelerating cognitive and social impairments. Special attention is given to high-level cognitive processes, including executive functions and social cognition, which are particularly vulnerable to these disruptions. Highlighting the interplay between IGF-I, neuroinflammation, and stress, this work underscores the need for affordable and accessible diagnostic tools and therapeutic strategies. This review contributes to a deeper understanding of IGF-I’s multifaceted role in AD, offering new insights for addressing the growing global burden of dementia.

## 1. Introduction

Alzheimer’s disease (AD) is one of the leading causes of dementia, or a loss of intellectual capacity, in the global population over sixty-five. Age, family history, and genetic background are the primary risk factors [1] in most AD patients with a genetic predisposition. However, a significant proportion of patients, around 95%, present with sporadic AD in contrast to familial AD [2]. This reveals that unidentified factors may modulate the development of pathophysiological mechanisms leading to the disease [3]. Sporadic AD underscores the existence of various multifactorial disorders at the genetic, environmental, inflammatory, and metabolic levels [2,4,5], which further complicates its assessment management. The report published by HelpAge International in 2015 [6] estimated that the population over sixty would reach 12.3%, implying that at least sixty-two countries could be categorized as hyper-aged societies. The studies have established that aging is advancing rapidly, but estimates remain scarce in middle- and low-income countries [7]. Despite the growing prevalence of neurodegenerative diseases, few countries are implementing appropriate early diagnosis and intervention strategies. According to the World Health Organization [8], fifty-five million people are living with dementia, with approximately 7.7 million new cases detected each year, of which nearly 60% reside in middle and low-income countries [8]. However, recent projections indicate that the prevalence in low and middle-income countries will increase by 47% to 71% over the next 15 years, compared to 23% in Europe and 41% in the US [9].

Early alterations in AD often go undiagnosed, or symptoms are misdiagnosed or ignored due to low specialization and wrong screening methods used in primary attention. Furthermore, early diagnosis could reduce the economic burden of treating these patients. Numerous studies have demonstrated the high socioeconomic costs of dementia. It is estimated that dementia costs the US economy USD 305 billion annually and the UK economy GBP 34.7 billion [10,11]. Considering the economic cost, a recent systematic review study [12] indicates that the cost of care and treatment for a patient with AD in low to middle-income countries hovers around USD 20,000. Due to the absence of reliable epidemiological studies and the disease burden in developing countries, economic costs will be underestimated or not thoroughly studied.

Currently, early detection largely relies on expensive or invasive procedures such as positron emission tomography for beta-amyloid (Aβ), known as Pittsburg PET [13], or biochemical analysis of cerebrospinal fluid (CSF) for the measurement of Aβ and Tau [14,15]; procedures that are not accessible to for low and middle-income countries. Thus, there is a need to explore alternative approaches that are remarkably affordable, sensitive, specific, and accessible blood-based biomarkers for low-resource populations. Additionally, it is essential to consider multifactorial biomarkers and their relationship with cognition. Neurocognitive endophenotypes are necessary for searching for these biological markers [16]. These refer to indicators that exhibit a specific relationship between cognitive processes and underlying neurophysiological or neurobiological function, which could shed light on new techniques or mechanisms involved in AD.

AD’s pathological features appear up to 20 years before (neurofibrillary tangles and Aβ plaques) [1]. However, the mechanisms of deterioration are not fully understood. This is one of the reasons why metabolic dysregulation in the brain has been proposed as an indicator of specific pathophysiological mechanisms in AD, potentially playing a crucial role in its early detection [17,18]. In addition to the interrelationship between dementia, non-communicable diseases, and lifestyle-related risk factors [19], it is known that these risk factors significantly affect middle-aged individuals. These risk factors include physical inactivity, imbalanced diets, tobacco and alcohol consumption, obesity, hypertension, and diabetes mellitus [20,21,22,23,24,25,26]. Other risk factors include social isolation, low educational attainment, cognitive inactivity, anxiety, and midlife depression [27,28,29,30,31,32]. It is interesting to note that these mid-to-late life risk factors are associated with lifestyle and behavior. These factors become even more relevant, considering that a considerable proportion of patients (more than 95%) have sporadic AD.

Studies over the last 20 years have linked AD and associated cognitive impairment to metabolic disruption related to insulin-like growth factor-I (IGF-I) peptides and other stress markers [5]. Furthermore, these lifestyles and risk factors are associated with insulin family peptides, especially IGF-I [5]. Over time, IGF-I’s absence, resistance, or loss could initiate various pathophysiological mechanisms in AD. These mechanisms include amyloid-beta (Aβ) clearance, oxidative stress, altered glucose metabolism, inflammation, and increased cell death [33]. Given this, lifestyle modification could, directly and indirectly, change insulin-associated signaling pathways, and in general, by modifying risk factors, we could promote a 40% reduction in the prevalence of dementia and AD [34].

On the other hand, cognitive impairments associated with AD are rare in middle age (20 years before the standard diagnosis of AD). This discrepancy can be explained using general cognitive measures such as the Mini-mental State Examination (MMSE), Montreal Cognitive Assessment (MoCA), or other brief cognitive screening tests, which are helpful in more characterized clinical samples but not in diverse and relatively young population-based participants, mainly due to the insufficient validation of these tests in these populations [35]. Another possibility is the ceiling effect of the tests, combined with the lower cognitive demands required for their completion. This is reflected in the tests’ low sensitivity and specificity when applied to middle-aged populations experiencing early cognitive changes. Furthermore, investigating how insulin-related metabolic disruption could be affected in middle age and its association with cognitive profiles would improve our understanding of AD or its use as a biomarker. Taking this into account, the use of new neurocognitive endophenotypes would have clinical utility, especially in less-studied cognitive impairments such as social cognition/theory of mind and executive function in AD, along with new biomarkers like IGF-I, stress, and neuroinflammation, analyzed together. We believe that metabolic disruption, the accumulation of stress risk, and neuroinflammation processes may precede the onset of clinical symptoms and trigger AD pathology, early evident in executive function and social cognition impairment.

### 1.1. Insulin-like Growth Factor 1

The IGF-I, initially called “somatomedin C”, is a neuropeptide composed of seventy amino acids with a molecular weight of 7.5 kDa. It is mapped to chromosome 12 in humans and chromosome 10 in mice [36,37]. This peptide shares a 48% structural homology with insulin and a 50% similarity with its precursor, proinsulin [36,38,39]. IGF-I is produced paracrinally and endocrinally by various tissues, including the brain, muscle, cartilage, pancreas, and others. However, its primary source is the liver, where 75-80% of the total available IGF-I is produced by hepatocytes and released into the bloodstream to reach various organs and cellular targets [40,41]. The production of IGF-I in the liver is centrally controlled by the hypothalamus, with the assistance of the pituitary gland, which, when stimulated, promotes the release of growth hormone (GH). GH, in turn, encourages the release of IGF-I into the bloodstream, where, aided by insulin-like growth factor binding proteins (IGFBP), it extends its availability, half-life, and transport to distant sites [42]. IGF-I binds with an affinity of approximately 98% to IGFBPs, ensuring its viability.

**Figure 1:**
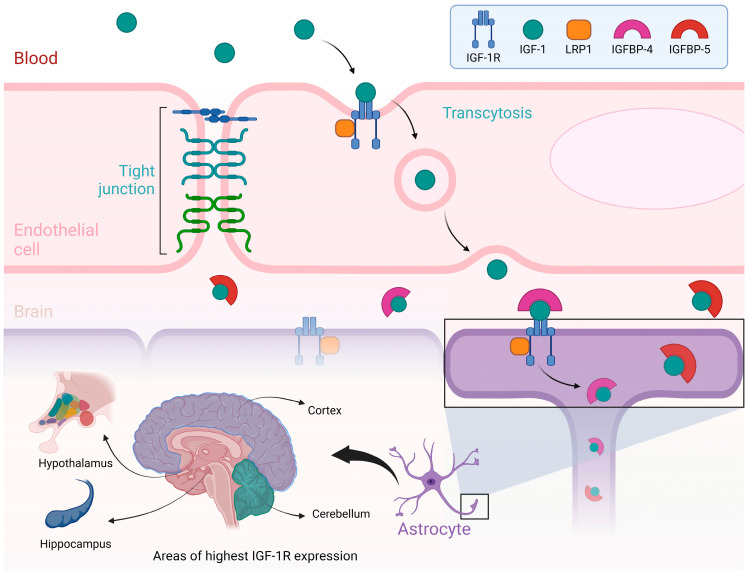
Transport of IGF-I from blood to brain.

**Note:** Figure 1 illustrates the process by which Insulin-like Growth Factor I (IGF-I) is transported from the bloodstream to the brain, emphasizing the role of the blood–brain barrier and the regions of the brain where IGF-IR are most abundantly expressed. At the top of the figure, a blood vessel is depicted as having an endothelial cell, a crucial part of the blood–brain barrier. IGF-I, circulating in the blood, binds to its receptor, IGF-IR, on the endothelial cell membrane. This IGF-I/IGF-IR complex is internalized through a process known as transcytosis, which helps the passage of IGF-I across the blood–brain barrier and into the brain. More proteins, including LRP1 and IGF-binding proteins (IGFBP-4 and IGFBP-5), also play crucial roles in modulating the availability and transport of IGF-I during this process. The lower part of the figure highlights the brain regions with the highest expression of IGF-IR, including the cortex, cerebellum, hypothalamus, and hippocampus. These regions are associated with essential functions such as growth regulation, metabolic control, and cognitive processes. The figure also depicts the interaction of IGF-I with astrocytes, which are glial cells vital for supporting and modulating neuronal function. Illustrations were created using BioRender (www.biorender.com, accessed on 23 August 2024).

Various binding proteins attach IGF-I to one of the six IGFBP, depending on the specific organs and tissues [42]. For instance, IGFBP-3 is the most abundant binding protein for IGF-I in the bloodstream, accounting for about 80% of all IGF-I binding to these proteins, forming a 1:1 molar ratio [43]. IGFBP also binds to IGF-I within the liver, allowing growth hormone to act on the liver to produce more IGF-I continuously. There are six IGFBPs, with IGFBP-2 being the most abundant in cerebrospinal fluid (CSF) [44]. IGFBP-4 and IGFBP-5 are highly expressed in the brain [45]. In the central nervous system (CNS), IGF-I performs a multitude of actions, including neurotrophic, neuromodulatory, and neuroendocrine functions, and it also operates during various stages of development [46]. For instance, it plays a role in cellular growth and development, differentiation, neurogenesis, synaptogenesis, and cytogenesis [36]. See Figure 1.

### 1.2. Insulin-like Growth Factor 1 Signaling Mechanisms

IGF-I exerts its physiological effects primarily by binding to its receptor, IGF-IR, but also using the insulin receptor (IR) due to its structural homology. IGF-IR and IR can also form hybrid heterodimeric receptors, which are present in various cell types throughout the body and exhibit specific biological functions depending on the tissue. These hybrid receptors have been identified in brain cells, which play critical roles in neuronal signaling and peripheral tissues such as muscle, liver, and adipose tissue [47]. IGF-IR is a tetramer composed of two extracellular α chains and two transmembrane β chains containing a tyrosine kinase domain [37]. The binding of IGF-I to its receptor originates from a cysteine-rich region of the α subunit of the receptor, triggering a conformational change that allows the activation of its tyrosine kinase domain, phosphorylating the corresponding sites on the β subunit [48].

On the other hand, IGF-IR remains in a catalytically inactive state when unphosphorylated until it binds to IGF-I, allowing receptor autophosphorylation. In addition to the activation of the external receptor on the membrane, IGF-IR activation also promotes the phosphorylation of various substrates in intracellular signaling, including one of the key ones, Insulin Receptor Substrate 1 (IRS-1) [48], but also others, such as Insulin Receptor Substrate 2 (IRS-2), Src Homology 2-Containing Protein 1 (SHC1) [44].

**Figure 2:**
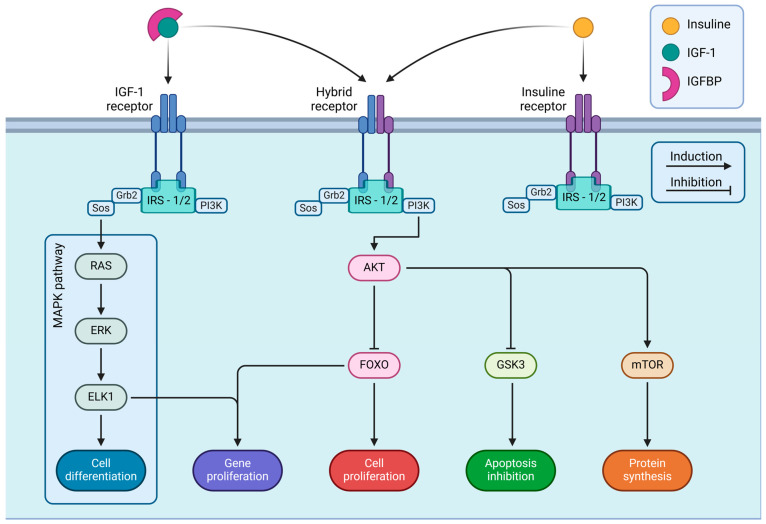
Intracellular signaling pathways of IGF-I and insulin receptors.

**Note:** Figure 2 details the intracellular signaling pathways triggered by IGF-IR and IR. These receptors are embedded in the cell membrane and become activated upon binding with their respective ligands, IGF-I and insulin, starting a series of signaling cascades that regulate various cellular functions. The figure shows three main types of receptors: IGF-IR, IR, and a hybrid receptor combining elements of both. Upon activation, each receptor associates with IRS-1 or IRS-2, which is essential for transmitting signals into the cell. The receptor-IRS complex contains two principal signaling pathways: the MAPK and PI3K/AKT pathways. IGF-I and insulin receptors integrate extracellular signals through these pathways to activate intracellular cascades, which determine cell fate and play critical roles in development, metabolism, and organismal homeostasis. Illustrations were created using BioRender (www.biorender.com, accessed on 3 January 2025).

Activating IRS-1/IRS-2 substrates is essential for receptor activation as they promote several intracellular signaling pathways [49]. Among the different IGF-I activated signaling pathways, the most well-studied ones involve the Phosphatidylinositol-3 Kinase (PI3K)-AKT Serine/Threonine Kinase 1 (AKT1)-Mammalian Target of Rapamycin (mTOR) pathway, and the GSK3 (Glycogen Synthase Kinase-3) inhibition pathway [50], the PI3K-AKT-Forkhead Box Protein O (FOXO) pathway, and the Mitogen-Activated Protein Kinase (MAPK) pathway [44]. The activation of these canonical pathways initiates intracellular signaling related to cell growth, metabolism, and apoptosis inhibition (PI3K pathway), as well as mitogenesis and cell differentiation (MAPK pathway) [36]. See Figure 2.

Traditionally, glucose uptake in the brain was considered insulin-independent, relying on constitutive glucose transporters such as GLUT1 and GLUT3. Nowadays, it is known that insulin modulates cerebral glucose metabolism through concerted action on astrocytes [51]. The access of serum IGF-I to the brain is mediated by the blood–brain barrier (BBB) at the choroid plexus and blood vessels [36]. One of the primary mechanisms for crossing the BBB is the process of transcytosis, where IGF-I is transported through endothelial or epithelial cells from the blood vessel lumen across the cell and subsequently released into interstitial space or CSF. The BBB regulates the flow of nutrients and metabolites from blood to the central nervous system (CNS). It controls their availability through different transport systems [52], including IGF-IR and low-density lipoprotein receptor-related protein 1/2 (LRP1, LRP2). Upon entering the CNS, IGF-I reaches the cerebrospinal fluid and areas of the parenchyma, such as the hypothalamus and hippocampus [53], and other regions, such as the cortex and cerebellum. As mentioned earlier, IGF-I plays an essential role in brain development, both prenatal and postnatal, mediating various cellular processes such as mitogenesis, cell growth, differentiation, and synaptogenesis. It also plays a role in the adult brain, participating in brain homeostasis, neurogenesis, and the response to brain trauma and pathology in general [54,55]. In recent decades, the study of the effects of IGF-I on the brain has been associated with aging, longevity, cognition, neurodegeneration, and neuropsychiatric disorders [41,56,57,58]. The IGF-I system comprises a complex regulatory network operating throughout the body with multiple neuroprotective properties [59].

### 1.3. Insulin-like Growth Factor 1 and Alzheimer’s Disease

Various experimental studies in rodents and studies involving human participants have demonstrated the relationship between IGF-I and cognition [56,60]. IGF-I plays a critical role in modulating multiple neuronal functions, including sleep homeostasis via hypothalamic orexinergic cells [61] and cognitive processes, which are influenced by sensory facilitation involving acetylcholine neurons and astrocytes in the cortex [62,63]. Additionally, IGF-I contributes to maintaining a healthy lifestyle, mainly through physical exercise and cognitively demanding activities such as environmental and cognitive enrichment [5].

Because of its multiple actions in the brain, including synaptogenesis, hippocampal neurogenesis, cell maturation, cell proliferation, and reduction of apoptosis, IGF-I is closely associated with healthy aging. Elevated serum levels of IGF-I have been linked to better cognition and overall brain health [36,64]. In this way, its relationship with healthy aging is often associated with elevated serum levels and better cognition. “Regarding AD, IGF-I deficiency results from extensive changes in brain function, closely resembling AD pathology and its associated comorbidities [33,65]. However, different studies show conflicting results regarding the relationship between IGF-I and AD [66], with some highlighting its positive effects in counteracting AD [67]. In contrast, other studies suggest that reducing IGF-I could be beneficial for increasing longevity [68], as well as differing views on its role in AD.

Different studies in preclinical and clinical settings have found that IGF-I levels in the brain largely depend on participant characteristics (gender differences, socioeconomic status, nutritional differences) and IGF-I/IGFBP serum levels crossing BBB [36], which is mainly variable depending on the study population and study settings. However, other studies show that insulin and IGF-I as treatments in neuronal cultures reduce the phosphorylation of APP [69]. In humans, there is evidence of the relationship between ApoE-ε4 and tertiles of IGF-I receptor-stimulating activity, with ApoE-ε4 homozygotes showing lower levels of IGF-I receptor-stimulating activity than heterozygotes and non-carriers [70]. Furthermore, low serum levels of IGF-I are associated with more significant atrophy and a higher risk of developing AD [67]. Thus, many pathologies related to AD are explained by serum IGF-I deficiency, such as cerebral amyloidosis, tau phosphorylation, inflammation, glucose uptake, cerebral blood flow, and others.

IGF-I levels in serum increase with environmental and cognitive enrichment, exercise, and healthy lifestyles, causing this peptide to cross the BBB to reach the brain, promoting healthy homeostasis in various neuronal systems, including the cholinergic system [63]. Although IGF-I decreases in serum during aging, healthy aging programs generally incorporate exercise in multiple forms (such as walking, yoga, running, or physical exercise) to promote health, leading to the entry of IGF-I into the brain [71]. It is also known that mentally demanding or active tasks promote brain stimulation, as does socialization, which supports brain health, aspects that are also related to the IGF-I peptide pathway in the brain [72,73,74,75]. For example, in mice, environmental enrichment has been associated with cognitive performance, social interaction, oxidative stress, and dopamine regulation, and it promotes the entry of IGF-I into the brain in these situations [76,77]. Thus, insufficient cognitive and environmental enrichment is a risk factor for cognitive and emotional disorders at any age [78,79]. On the other hand, its supplementation/increase at any age and exercise would improve cognitive performance through the neuroprotective role of the IGF-I peptide.

Finally, numerous studies confirm the relationship between IGF-I serum levels and cognitive performance, specifically in older adults and assessments of executive function, selective attention, and working memory. This cognitive profile was obtained using MMSE, Trail Making Test A and B scores, Ruffs 2 and 7 Test, and number and letter sequencing [80]. In individuals with mild cognitive impairment, it has been found that IGF-I is correlated with better cognitive performance after adjusting for insulin levels, body mass index, and age [81]. Regarding women, research has shown that insulin, a member of the insulin family with 48% structural homology to IGF-I, is negatively associated with executive functioning. Specifically, higher levels of insulin resistance were found to correlate with poorer performance in the executive function domain [82].

### 1.4. Social Interaction and Insulin-like Growth Factor 1

IGF-I plays a crucial role in cognition by supporting essential neuronal functions such as synaptic plasticity, neurogenesis, and synaptic modulation within the central nervous system (CNS). As previously mentioned, IGF-I increases its entry into the brain through environmental enrichment, which, in studies with mice, has been primarily linked to physical exercise and enhanced social interaction with other mice of both sexes [41]. These effects have been mainly studied in animal models. IGF-I promotes neuronal survival, synaptic plasticity, and neurogenesis in crucial areas, such as the prefrontal cortex, critical for cognition and social behavior [83,84,85].

Initial studies derived from animal models of conditions such as schizophrenia and depression suggest a deficit in neurotrophic factors like IGF-I and Brain-Derived Neurotrophic Factor (BDNF) [83]. Transgenic models of these disorders, characterized by reduced social interaction and repetitive behaviors, have shown that the administration of IGF-II can reverse these behaviors [84], emphasizing the importance of IGF-related pathways in regulating social interaction. Models of mice lacking IR or IGF-IR in astrocytes exhibit impaired social novelty. These findings suggest that IR and IGF-IR in astrocytes play differential roles in modulating social behavior’s cognitive and motivational components [85].

Moreover, IGF-I’s modulation of excitatory synaptic transmission in pyramidal prefrontal cortex neurons highlights its influence on cognitive processes that underlie social interactions [86]. Animal studies demonstrate that IGF-I, through neurogenesis and synaptogenesis, supports the formation and maintenance of neural circuits involved in social behavior [87]. In human studies, the role of IGF-I in sociability and social cognition has not been extensively explored in adults. Still, deficits related to IGF-I have been observed in other neuropsychiatric disorders [58]. Deficits in the somatotropic axis and IGF-I have been linked to impairments in social cognition and interaction, as assessed using tests like the Schedules for the Assessment of Social Intelligence (SASI), which involve tasks related to facial emotion recognition and theory of mind [88].

Furthermore, in older adults, the decline in IGF-I associated with aging has been linked to increased loneliness, depression, and social isolation, with prevalence rates ranging from 16% to 32% [89]. This supports the connection between IGF-I reduction and aging-related social impairments, as IGF-I also modulates higher cognitive functions such as empathy and emotional recognition, both crucial for effective social interactions [56,90]. Nonetheless, evidence suggests that prolonged treatments with IGF-I, IGF-II, and other neurotrophic factors like BDNF could represent promising therapeutic approaches for neuropsychiatric disorders [91,92,93] and neurodegeneration [94,95,96,97].

### 1.5. Other Mechanisms Involved in Alzheimer’s Disease and Insulin-like Growth Factor 1

Key processes involved in Alzheimer’s disease include impaired β-amyloid clearance, increased oxidative stress, heightened neuroinflammation, and chronic stress. The present work is focused on markers of neuroinflammation and stress, as these can be readily measured in the laboratory using blood markers such as interleukins or TNF-α, and they directly affect the IGF-I signaling pathway [98].

On the other hand, stress is a normal part of people’s lives that can become elevated, especially in Western societies. It has been observed that the normal glucose response is altered in response to emotional stress, which is also related to increased anxiety and depression in normal individuals and those with diabetes [99]. IGF-I is altered in stressful situations due to the action of glucocorticoids, affecting the IGF-I signaling pathway both prenatally and postnatally [100]. This pathway is linked to the alteration of IRS-1, which elevated glucocorticoid levels and neuroinflammatory factors such as TNF-α can inhibit. These elements activate the JNK (c-Jun N-terminal kinase) pathway, resulting in IGF-I resistance and disrupting its signaling [98], ultimately leading to actions that oppose the effects of IGF-I. Chronic stress and post-traumatic stress can affect insulin family peptides [101]. Chronic stress and increased glucocorticoids would promote resistance to IGF-I in areas such as the hippocampus and hypothalamus, blocking its neurotrophic effects; a similar result would occur due to stress on the insulin pathway [102]. It is also known that stress can alter liver function, affecting metabolic functions associated with insulin-like peptides or directly inhibiting the IGF-I pathway by affecting IRS-1 [103]. Even more interesting is that this resistance effect can be generated acutely [104].

## 2. Link Between Mechanisms Contributing to AD and IGF-I Signaling Pathways

### 2.1. Alzheimer’s Disease, Neuroinflammation, and Stress

**Figure 3:**
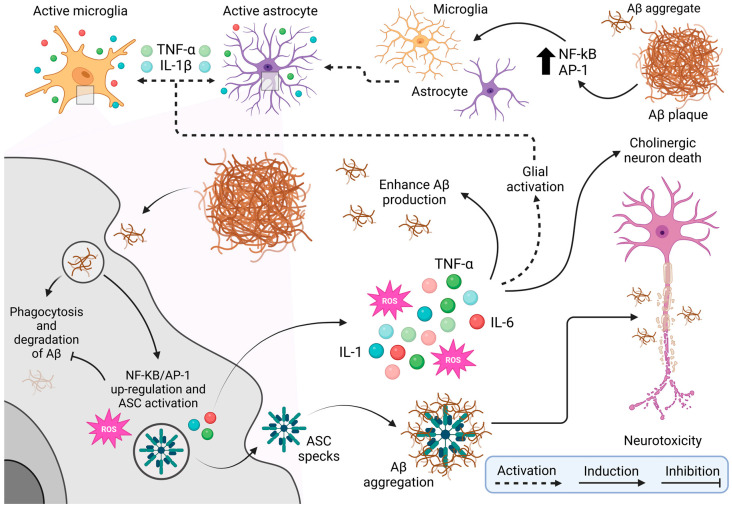
Cellular and molecular mechanisms of neurotoxicity in Alzheimer’s disease.

**Note:** Figure 3 provides a comprehensive schematic of the cellular and molecular mechanisms underlying neurotoxicity associated with beta-amyloid (Aβ) plaque accumulation, a hallmark of neurodegenerative diseases such as Alzheimer’s disease. The figure begins by showing the activation of microglia, the brain’s resident immune cells, in response to Aβ presence. This activation is mediated by transcription factors such as NF-kB and AP-1, which drive a proinflammatory state. Astrocytes, another type of glial cell, are similarly activated by inflammatory signals, including cytokines like TNF-α (tumor necrosis factor-alpha) and IL-1β (interleukin 1 beta) released by microglia. This glial activation, mediated by NF-kB and AP-1, perpetuates inflammation within brain tissue. Microglia try to clear Aβ plaques through phagocytosis, but this process is often incomplete or ineffective, leading to Aβ accumulation. Persistent interaction with Aβ upregulates NF-kB/AP-1 expression and activates the ASC (apoptosis-associated speck-like protein holding a caspase recruitment domain) complex, which generates reactive oxygen species (ROS) and releases more pro-inflammatory cytokines such as IL-1, TNF-α, and IL-6. The ROS production, combined with ongoing inflammation, worsens oxidative stress, further aggravates Aβ aggregation and promotes a toxic environment detrimental to cholinergic neurons. These neurons, crucial for memory and learning, are especially vulnerable to Alzheimer’s disease. The resulting neurotoxicity and later death of cholinergic neurons drive the cognitive decline characteristic of the disease, creating a vicious cycle of chronic inflammation, neuronal damage, and worsening pathology. Illustrations were created using BioRender (www.biorender.com, accessed on 3 January 2025).

Neuroinflammation and chronic stress are significant contributors to the progression of Alzheimer’s disease (AD). Neuroinflammation is characterized by persistent activation of microglia and astrocytes, which release proinflammatory cytokines such as IL-1, IL-6, and TNF-α. This chronic activation exacerbates the accumulation of Aβ plaques and hyperphosphorylated Tau, driving neuronal dysfunction, cognitive impairment, and sleep disturbances [105,106,107,108,109]. Microglial attempts to clear Aβ plaques through phagocytosis are often incomplete, leading to further accumulation and increased oxidative stress, which promotes a toxic environment detrimental to cholinergic neurons [107,108,109,110]. This vicious cycle contributes to the progressive cognitive decline characteristic of AD [4]. See Figure 3.

**Figure 4:**
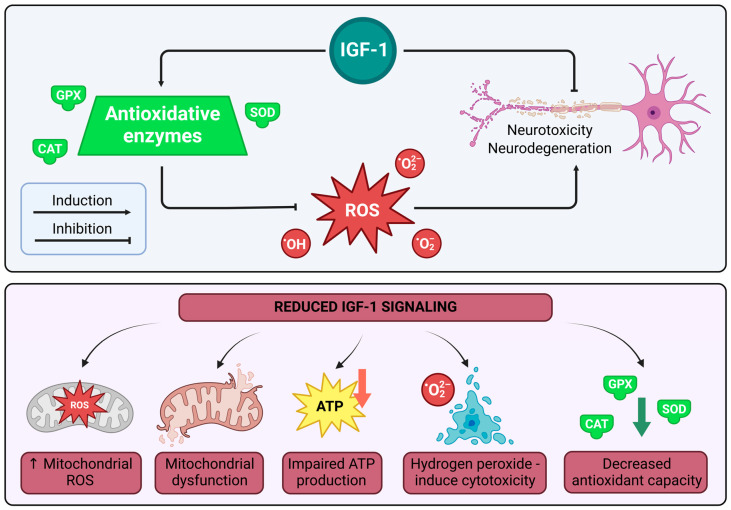
IGF-1-mediated regulation of ROS and oxidative stress in neurodegeneration.

**Note:** Figure 4 illustrates IGF-1’s role in oxidative stress regulation. Under normal conditions, IGF-1 enhances antioxidative enzymes—superoxide dismutase (SOD), catalase (CAT), and glutathione peroxidase (GPx)—converting superoxide radicals (O_2_^−^) and hydrogen peroxide (H_2_O_2_) into water, reducing reactive oxygen species (ROS) production and preventing neurotoxicity and neurodegeneration in neurons via induction of these enzymes. Conversely, reduced IGF-1 signaling decreases antioxidant capacity, causes mitochondrial dysfunction, impairs ATP production, and elevates mitochondrial ROS, increasing vulnerability to hydrogen peroxide-induced cytotoxicity and exacerbating neuronal damage. The diagram highlights IGF-1’s protective effect, with evidence linking its deficiency to heightened ROS and neurodegeneration, while overexpression mitigates oxidative damage. Illustrations were created using BioRender (www.biorender.com, accessed on 3 January 2025).

IGF-1 plays a crucial role in oxidative stress (OS) regulation by enhancing superoxide dismutase (SOD) activity, which catalyzes the conversion of superoxide radicals into less harmful molecules such as hydrogen peroxide, further reduced to water by catalase (CAT) and glutathione peroxidase (GPx) to prevent oxidative damage and ROS production [111]. Reduced IGF-1 signaling is associated with decreased antioxidant capacity, mitochondrial dysfunction, impaired ATP production, elevated mitochondrial ROS, and increased vulnerability to hydrogen peroxide-induced cytotoxicity [112]. Additionally, IGF-1 supports neuronal homeostasis under oxidative stress conditions, particularly during axonal degeneration [113,114]. Neurodegeneration models consistently demonstrate a link between IGF-1 deficiency and heightened oxidative stress responses, while IGF-1 overexpression correlates with reduced oxidative stress [115]. See Figure 4 below.

**Figure 5:**
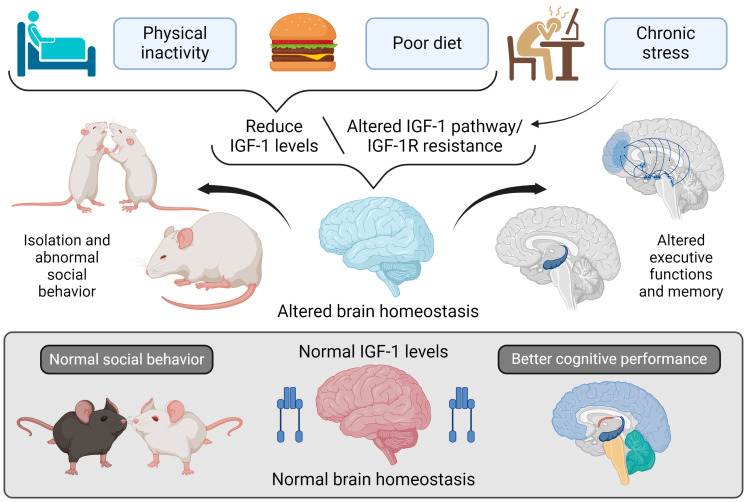
Impact of lifestyle on IGF-I regulation and brain function.

**Note:** Figure 5 explores the relationship between lifestyle factors and regulating IGF-I in the brain and its effects on brain homeostasis, social behavior, and cognitive functions. At the top of the figure, three lifestyle factors are shown that can lower IGF-I levels or disrupt its signaling pathway: physical inactivity, poor diet, and chronic stress. These factors contribute to reduced IGF-I levels or IGF-IR resistance, impairing normal IGF-I signaling in the brain. This disruption in IGF-I signaling adversely affects brain homeostasis in two keyways: 1. Altered Social Behavior: Mice with diminished IGF-I levels show social withdrawal and abnormal social interactions, reflecting dysfunction in brain areas responsible for social behavior. 2. Impaired Executive Functions and Memory: Reduced IGF-I signaling, or IGF-IR resistance, also adversely affects executive functions and memory, as depicted by changes in critical brain regions such as the hippocampus and prefrontal cortex.

Systemic factors such as obesity, hypothalamic dysfunction, and lifestyle-related elements, including diet, exercise, and sleep patterns, interfere with normal immune responses and exacerbate neurodegeneration [110]. Interestingly, many of these factors are associated with cardiovascular health and metabolic regulation, where insulin-like peptides, particularly IGF-I, play a crucial mediating role [5,116]. See Figure 5. IGF-I has been shown to modulate inflammatory pathways and reduce the pathological effects of TNF-α, with treatments like Cerebrolysin demonstrating benefits in AD patients by increasing IGF-I levels and mitigating inflammation [116,117].

In contrast, the lower section of the figure illustrates the benefits of supporting normal IGF-I levels. Mice with healthy IGF-I levels display normal social behavior and maintain brain homeostasis, which is essential for the best cognitive performance. Illustrations were created using BioRender (www.biorender.com, accessed on 23 August 2024).

Stress further amplifies these neuroinflammatory responses. Chronic psychological stress and traumatic experiences are associated with increased Aβ production, cortical atrophy, and cognitive deficits [118]. Epidemiological studies have also highlighted higher incidences of anxiety, depression, and other neuropsychiatric disorders as comorbidities in AD and mild cognitive impairment (MCI) [119,120,121,122,123,124]. Elevated glucocorticoids during chronic stress impair IGF-I signaling by activating pathways such as JNK, which disrupts the protective effects of IGF-I and promotes neuroinflammation [65,98,125].

In animal and human studies, IGF-I deficiency has been associated with behavioral disturbances, reduced sociability, and increased vulnerability to stress. IGF-I also modulates resilience mechanisms, with FKBP5—a glucocorticoid receptor co-chaperone—positively correlated with reduced IGF-I levels and epigenetic dysregulation in stress response pathways [125,126,127,128]. This interplay between stress, neuroinflammation, and IGF-I signaling highlights the importance of addressing lifestyle factors and implementing targeted interventions to mitigate AD pathology.

### 2.2. Executive Functions (EF) in Alzheimer’s Disease

After considering some pathological mechanisms of AD related to metabolism, stress, and neuroinflammation, we want to mention the possible cognitive endophenotypes associated with highly hierarchical brain behavior that are underestimated in brief cognitive test studies. The EF is one of the main cognitive functions constantly tested in human life interactions, considering social and psychological adaptability to the context, stressful life events, environmental stressor coping and resilience, decision-making, organization, and behavior monitoring [129]. Therefore, EFs are a good indicator of the brain’s ability to adapt to new environmental changes through the aging process because the higher-level cognitive ability of EF controls and coordinates other cognitive skills, which can be sensitive to cognitive impairments.

Activities of daily living (ADL) require lower levels of EF, and some cognitive tests have a moderate relationship with ADL, such as the clock-drawing test, the trail-making test, the Stroop test, and verbal fluency [130]. Considering this, EFs are not thoroughly assessed, and impairments might go unnoticed in preclinical participants or routine clinical evaluation. Brief cognitive tests have shown that 32–42% of older participants with a typical MMSE score exhibit impaired EF [131], and subsequent EF impairment in older adults and participants with AD increases by 64% or more [132]. Recent studies reveal a possible overlapping between memory impairment and EF, which might hinder an accurate differential diagnosis from cognitively unimpaired to pathological aging associated with AD [133,134,135]. Monitoring working memory (WM) during aging reveals a reduction in preclinical and clinical stages (MCI and AD). Therefore, monitoring performance in working memory and executive function may indicate the progression from normal cognition to MCI to Alzheimer’s disease [136]. Elevated levels of IGF-1 have been associated with improved executive function and attention in individuals with mild cognitive impairment (MCI) and Alzheimer’s disease (AD). In contrast, higher baseline IGF-1 levels were linked to better performance on executive function tasks, such as the Trail Making Test B (TMT-B), in AD patients, indicating a potential protective effect of IGF-1 against cognitive decline [137]. Furthermore, a lower serum IGF-1/IGFBP-3 molar ratio has been connected to poorer executive function in MCI patients, particularly those with type 2 diabetes mellitus (T2DM), suggesting that IGF-1 may play a role in the development of cognitive impairment [138].

Participants with typical AD show EF deficits like those in Frontotemporal Dementia (FTD). However, their qualitative profile differs, with a more attentional/switching pattern in FTD and more working memory in AD. On the other hand, pure amnestic AD conditions did not show EF deficits [132]. Neuroimaging analysis shows that typical AD exhibits more temporoparietal atrophy than frontal, indicating the relation of posterior projections to EF [132]. A new study in mild cognitive impairment (MCI) patients with positive biomarkers for AD shows that a worse performance in EF could help to discriminate between possible AD diagnosis and other conditions [133]. Their result suggests that executive and memory functions are impaired in possible AD (CSF+), and the organization of the material during a memory task is the factor that helps to distinguish the profile. The relationship between EF scores and CSF concentrations in AD patients was also described by van der Vlies et al. [135], who reported that participants with worse memory performance, mental speed, and EF exhibit low levels of Aβ-42 and extremely high CSF levels of Tau and p-tau. Abellán Martínez et al. [134] stated that the typical reduction of cognitive performance in memory tasks throughout the aging process, particularly in participants with possible AD, may stem from executive function impairment rather than primary memory deterioration. In this sense, good episodic memory test performance could be conditioned by promising access to EF resources in MCI patients. Considering the EF performance during routine evaluation could lead to more precise/early diagnosis and intervention in patients with MCI.

Regarding the relationship with IGF-I, some studies describe an association with EF. Higher IGF-I levels were related to better scores in verbal fluency in healthy aging [139], with a similar tendency in global cognitive performance in MCI patients [81]. One possible explanation of these results is the moderation role of IGF-I in brain homeostasis, where the disruption may alter cognitive function.

### 2.3. Social Cognition in AD

On the other hand, other high-level cognitive functions are the theory of mind (ToM) and social cognition, which help build mental representations of the social world and enable interaction with representational identities, objects, and people under various environmental and situational conditions. Many environmental features in each social context contain high emotional and stressful content. From early childhood, the development of the mind allows children to recognize mental states with different complexity, differentiate between representational objects and symbols from animated entities, and understand intentions, thoughts, and emotions [140]. This ability is the cornerstone of social cognition and develops throughout life, allowing us to interact socially appropriately during youth and adulthood. This essential process integrates with others to shape the social cognition system. In the real world, this integrated system allows people to interact with others. In AD research, some studies indicate that the theory of mind is affected in AD but less widespread than in FTD [141]. In addition to AD, patients exhibit social withdrawal, apathy, and disinterest in social interactions [121,123,142,143,144], and in some cases, these signs represent the first observable characteristics [145]. Patients with AD also alter cognitive empathy compared to cognitive and emotional empathy in FTD [146] and emotional processing [147]. Social cognition is separate from general cognition in AD [148] but probably shares some processes with executive functions, such as social working memory [140]. In addition, patients with AD show specific deficits in ToM processing, [149] decreased facial recognition ability, and poorer activation of the default mode network [150]. We believe these two high-level processes mediate the response to chronic stress, coping, and lifestyles and are essential modulators of brain metabolism.

In studies with animal models, IGF-I deficiency has been observed to lead to deficits in social behaviors. For example, mice with reduced levels of IGF-I exhibit problems in socialization manifested as less frequent and shorter interactions with other mice. IGF-I is critical for developing normal social behaviors [65,85]. Additionally, IGF-I may affect how individuals respond to social stress. Research has shown that stress can alter IGF-I levels, and these changes may be associated with alterations in social interaction [151]. For instance, chronic stress can reduce IGF-I levels in the brain, potentially contributing to abnormal social behaviors.

It is important to consider that serum levels of IGF-1 do not necessarily correlate with its levels in the brain [152]. This may explain the contradictory findings in human studies, where increased serum IGF-1 is sometimes associated with improved brain health and cognition [56,153,154,155], while in other cases, it is not [156,157,158,159]. This discrepancy could depend more on the degree of brain sensitivity or resistance to IGF-1 rather than on circulating levels. These observations highlight the need for new considerations in studying IGF-1 and its effects on cognitive and metabolic function.

Furthermore, IGF-I has been investigated in the context of neuropsychiatric disorders that affect social interaction, such as autism and schizophrenia [58]. Some studies suggest that alterations in IGF-I signaling may be involved in the pathogenesis of these disorders and that modulation of IGF-I could be a potential therapeutic avenue [90].

### 2.4. Diagnostic Tools for Alzheimer’s Disease

Diagnosing Alzheimer’s disease (AD) relies on innovative tools in high-income countries yet remains a significant challenge in low- and middle-income countries (LMICs), where limited access to advanced technologies hinders early detection. Neuroimaging methods, such as Pittsburgh PET and MRI, effectively identify amyloid-beta (Aβ) plaques and brain atrophy but are prohibitively expensive and inaccessible, especially in LMICs, where 60% of global dementia cases occur [8,13]. Cerebrospinal fluid (CSF) analysis, which measures Aβ42, total Tau, and phosphorylated Tau (p-tau), offers early insights into AD pathology but is invasive and impractical for widespread use [14,15]. Blood-based biomarkers, including plasma Aβ, neurofilament light (NfL), and p-tau isoforms (e.g., p-tau181, p-tau217), have emerged as promising alternatives due to recent advancements enabling non-invasive measurement with high diagnostic accuracy, correlating with amyloid and tau PET findings and predicting cognitive decline [109]. Similarly, lower serum IGF-I levels are associated with increased AD risk and hippocampal atrophy [67]. However, serum IGF-I does not reliably mirror brain activity due to variable blood–brain barrier (BBB) penetration and cerebral sensitivity, potentially explaining inconsistent cognitive benefits observed with elevated levels [152,153,154,155,156]. This underscores that brain responsiveness to IGF-I, modulated by neuroinflammation or receptor resistance, may outweigh circulating concentrations in determining its efficacy.

Integrating IGF-I with these diagnostic approaches offers the potential for improving AD management in resource-constrained settings. Elevated serum IGF-I enhances systemic metabolic function, yet its central effects are limited, necessitating direct assessment of brain activity. Non-invasive techniques like electroencephalography (EEG) and magnetoencephalography (M/EEG) address this need [160], leveraging the activation of the brain cortex by IGF-1 to record neurophysiological signals that characterize hallmarks of healthy and pathological aging [161], including early AD-related changes [35]. Complementing these tools, cognitive assessments such as the Mini-Mental State Examination (MMSE) and Trail Making Test, when paired with IGF-I monitoring, support diagnosis and prognosis, with higher IGF-I levels linked to improved executive function in mild cognitive impairment (MCI) [80,137,138]. This multifaceted strategy could reduce the economic burden of AD care, facilitate timely interventions, and advance equitable diagnostics globally.

## 3. Conclusions

Alzheimer’s disease is a leading cause of dementia, with over 95% of cases being sporadic and influenced by genetic, environmental, inflammatory, and metabolic factors. The intricate interplay between IGF-I, metabolic dysregulation, and lifestyle factors presents a critical yet underexplored opportunity to redefine AD management. Early detection methods, such as PET scans and CSF analysis, are often expensive and invasive, limiting accessibility, particularly in low-resource settings. This highlights the urgent need for affordable, sensitive, and specific blood-based biomarkers for early AD detection.

IGF-I is a key neuropeptide involved in neurogenesis, synaptogenesis, and neuroprotection, playing a crucial role in cognitive function and healthy aging. IGF-I deficiencies are associated with cognitive and social behavior deficits and have been implicated in neuropsychiatric disorders. Its role in AD is complex, with metabolic dysregulation—particularly involving insulin resistance and IGF-I signaling—proposed as a significant pathogenic mechanism. Disruptions in these pathways contribute to impaired amyloid-beta clearance, glucose metabolism dysfunction, oxidative stress, inflammation, and neuronal death.

Moreover, lifestyle factors such as physical inactivity, poor diet, and chronic stress significantly impact these metabolic pathways and may accelerate AD progression. Addressing these modifiable risk factors could reduce dementia prevalence and delay AD onset. Investigating the relationship between IGF-I, cognition, and social interaction may pave the way for more effective biomarkers and early interventions, ultimately transforming AD diagnosis and prevention strategies.

## Data Availability

No new data were created or analyzed during this study. Data sharing does not apply to this article.

## Abbreviations

Aβ	Amyloid Beta
AD	Alzheimer’s Disease
AKT	Serine/Threonine Kinase 1
BBB	Blood-Brain Barrier
BDNF	Brain-Derived Neurotrophic Factor
CSF	Cerebrospinal Fluid
EEG	Electroencephalography
FOXO	Forkhead Box Protein O
GH	Growth Hormone
GSK3	Glycogen Synthase Kinase-3
IGF-I	Insulin-Like Growth Factor 1
IGFBP	Insulin-Like Growth Factor Binding Proteins
IR	Insulin Receptor
IRS	Insulin Receptor Substrate
MAPK	Mitogen-Activated Protein Kinase
MCI	Mild Cognitive Impairment
MMSE	Mini-Mental State Examination
mTOR	Mammalian Target of Rapamycin
PET	Positron Emission Tomography
PI3K	Phosphatidylinositol-3 Kinase
TMT-B	Trail Making Test B
TNF-α	Tumor Necrosis Factor-alpha
